# An online survey of Malaysian long-term e-cigarette user perceptions

**DOI:** 10.18332/tid/118720

**Published:** 2020-03-30

**Authors:** Surajudeen A. Abdulrahman, Kurubaran Ganasegeran, Chin W. Loon, Abdul Rashid

**Affiliations:** 1 Department of Public Health Medicine, RCSI & UCD Malaysia Campus, George Town, Malaysia; 2 Health Education England, Fulbourn, United Kingdom; 3 Clinical Research Center, Seberang Jaya Hospital, Seberang Perai, Malaysia; 4 Department of Environmental Health and Licensing, City Council of Penang Island, George Town, Malaysia

**Keywords:** e-cigarette, long-term e-cigarette usage, vapers, tobacco harm reduction, Malaysia

## Abstract

**INTRODUCTION:**

The use of e-cigarettes (EC) has reached alarming proportions among Malaysians. On a national level, little is known about the profile and perceptions of Malaysian EC users. This study aimed to explore the prevalence of long-term EC usage and its associated factors among EC users in Malaysia.

**METHODS:**

This nationwide online questionnaire survey was administered among 694 EC users across 13 states and 1 Federal Territory in Malaysia, between January and April 2018. A survey link was e-mailed to EC users that were recruited from an official national vape entity through their Facebook association page. We obtained information on respondents’ sociodemographic characteristics, smoking habits, long-term e-cigarette usage and perceptions of EC use. We estimated long-term EC user prevalence and fitted multivariate regression models to predict factors associated with long-term EC usage. Statistical significance was set at p<0.05.

**RESULTS:**

Respondents were predominantly Malays (87.6%), aged >30 years (68.1%) and tertiary educated (71%). The majority were employed (93.1%) with a monthly household income of MYR 4000 or less (56.6%). About 84% were former smokers, while 10% were current smokers. The prevalence of long-term EC usage in this study was 82.3%. Most users believed that EC had helped them to cut down tobacco smoking (94.8%), reduced the urge to smoke (88.3%) and ultimately helped them to quit smoking (87.2%). Respondents aged >30 years and those who perceived that EC has helped them stop smoking were significantly more likely to be long-term EC users.

**CONCLUSIONS:**

Most respondents engaged in EC use to quit smoking. They were more likely to be long-term EC users if they were older and perceived that EC has helped them to quit smoking. This information is valuable for targeted prevention, health promotion and policy regulations.

## INTRODUCTION

Electronic cigarettes, also called e-cigarettes (ECs) or electronic nicotine delivery systems (ENDS), are a nomenclature of battery-powered devices with a heating element that produces inhalable aerosols^1^. These devices preclude combustible elements, propagating assumptions that they have lower toxic effects compared to conventional cigarette smoking^2^. With polarised commercialisation, global sales rose rapidly, invoking conventional smokers to become EC aficionados^3^. International estimates found that EC use grew from 2.3 million users in 2013 to 5.1 million in 2015^4^. From the Asian perspective, Malaysia emerged as a growing EC industry, projecting a million people as regular EC users^5,6^.

The successful marketability of ECs was due to the perception that ECs would supplement tobacco harm reduction^7^. Two previous studies have illustrated the consensus on harm reduction from EC use. The first was the recent study report on the public health consequences of ECs, which highlighted that there was conclusive evidence that complete substitution of ECs for combustible tobacco cigarettes reduced users’ exposure to numerous toxicants and carcinogens present in combustible tobacco cigarettes^1^. Another conclusive report highlighted that vaping poses only a small fraction of the risks of smoking and switching completely from smoking to vaping conveys substantial health benefits over continued smoking^8^. Although most literature has highlighted awareness and global trends of EC use, little has been explored about the relative risks and potential benefits of long-term usage^9^. The paucity of high-quality randomised controlled trials and meta-analyses has failed to establish the exact role and/ or magnitude of the contribution of ECs to tobacco harm reduction^10^. Few hypothetical inconsistencies emerged from preliminary investigations of EC use; while some public health advocates argued that ECs would help adults quit smoking^11–13^ others fear the potential for renormalisation of smoking^5^ that could impede tobacco control efforts^7^.

Although studies have reported that EC users perceived that ECs could alleviate smoking addiction, the extent to which ECs help smokers to quit remains unclear. One study, which assessed quit rates among quitline callers, found that those who used ECs for at least one month had higher quit rates compared to those who did not use ECs for a month^14^. However, that study was limited by the threshold of one month, which is generally not regarded as long-term when considering EC use^15^. Another recent investigation, a 2-year longitudinal follow-up study in the US, found significantly higher tobacco quit attempts and cessation rates among EC users^15^. A prospective 3.5-year observational study among a cohort of 9 never smokers who used ECs daily found no significant harmful health effects from EC use^16^. This study was, however, limited by its relatively small sample size. One new high quality randomised controlled trial concluded that ECs were more effective for smoking cessation than nicotine-replacement therapy when both products were accompanied by behavioural support^17^.

EC users believe that ECs can assist conventional smokers in reducing smoking, alleviating nicotine cravings and preventing relapse following smoking cessation^18^. A recent Malaysian study that explored reasons for EC use among youths and young adults, in Kuala Lumpur and Selangor, found that most EC users: use them to quit smoking; believe that ECs are not as harmful as conventional tobacco cigarettes and perceived that EC use is much healthier; and, cheaper and compatible for public open-space usage^19^. The variations of study designs, participant selection and small sample size pose substantial challenges to integrate and extrapolate findings from these studies. The Malaysian National E-Cigarette Survey (NECS) 2016 was a nationwide representative survey conducted among 4288 individuals by the Institute of Public Health (IPH), Ministry of Health Malaysia. NECS 2016 aimed to determine the prevalence of ever use, current use and factors associated with EC use amongst Malaysian adults. The survey utilised a complex sampling design to represent 19 million household Malaysian adults stratified by states and urbanity. Data from the NECS 2016 are available upon request to the Director General of Health, Ministry of Health Malaysia^20–22^. Based on the findings from the NECS 2016, the overall prevalence rate of EC use in Malaysia was 3.2%^21,22^. It was further reported that former EC users (users that used EC but stopped in the previous 30 days) found that ECs were either not satisfying or did not help in quit smoking initiatives^21^. Although these nationwide estimates of EC use were reported, there were no available data on long-term EC usage and associated user perceptions in Malaysia. The current study was a nationally representative survey that aimed to explore the prevalence of long-term EC usage and its associated factors among EC users in Malaysia.

## METHODS

### Study des ign and participants

Participants were adult men aged ≥18 years, chosen because of the high prevalence of e-cigarette use in Malaysia^10,22^. This exploratory online survey approached Malaysian EC users from the Malaysian Organization of Vape Entity (MOVE), a nationally representative entity that comprised 13485 registered EC users at the time of the study. EC users were approached through an official online Facebook social media page owned by MOVE. With permissions from the president and the administrator of the MOVE Facebook page, an announcement was created and hosted at the Facebook interface between January and April 2018. EC users who wished to participate in the study were required to fill in basic demographic details on a Google-form that consisted of name, age, gender, e-mail address and telephone contact number. A total of 4581 EC users volunteered to participate.

We estimated sample size with Stata 13.0 using the formula for one proportion, with the following parameters: P=1.4% which was the prevalence of EC use among the adult population in Kuantan^23^; α=0.05; and Power=90%. To determine a difference of 1.8% in the prevalence of EC use in our study population, we estimated that a sample size n=642 was required. We accounted for a 10% non-response rate and obtained a total sample size of 707.

A sampling frame that consisted of a list of 4581 volunteered EC users was used to randomly select the required number of participants in our study. Of these, 931 participants were excluded as there was no e-mail address or telephone number provided on the basic demographic information form. From the remaining 3650 participants, selection was done through a systematic sampling method, with an interval of 5 participants, until the sample size of 707 was reached. In April 2018, an e-mail was sent to these participants that comprised consent forms, study information and a survey link. Participants were required to reply to the e-mail with the completed consent form within two weeks. Responses of the survey were verified when consent forms were received. Ethical approval was obtained from the Joint Penang Independent Ethics Committee (approval number: JPEC 17-0035).

The survey was answered using a Google-form link. To prevent redundancy of participation, respondents personal identity number and demographic information were checked and collected based on time-stamped validation by the site administrator. If this was unclear, response veracity was confirmed through a telephone call to the participant contact number. Ten responses were rejected as we were unable to ensure response veracity and no consent forms were returned. Three female participants were excluded. In total, we received a verified nationwide response from 694 e-cigarette users (response rate 98.1%) across all 13 states and 1 Federal Territory in Malaysia: Johor (81), Kedah (23), Kelantan (12), Federal Territory of Kuala Lumpur (120), Melaka (27), Negeri Sembilan (22), Pahang (31), Perak (33), Perlis (5), Pulau Pinang (28), Selangor (231), Terengganu (19), Sabah (23), and Sarawak (39).

### Baseline definitions

Long-term EC usage had a threshold of ≥2 years, in accordance with recent prospective studies^15,16^. A current smoker was defined as an adult who smoked 100 cigarettes in his lifetime and who currently smokes conventional cigarettes^24^. Former smoker was defined as an adult who had smoked at least 100 cigarettes in his lifetime but who quit smoking at the time of the study^24^.

### Study instrument

We developed a structured, closed-ended, self-administered online questionnaire in local language based on current literature. The questionnaire was pilot tested among 10 EC users who were not included in the analysis. Detailed wording was moderated using feedback from these knowledgeable EC users. The final questionnaire consisted of 2 sections with 19 items. The first part assessed EC user profiles that included 9 items on sociodemographic characteristics: age, dichotomized as ≤30 years and >30 years; ethnicity categorised as Malays or non-Malays; education level categorised as tertiary education, or secondary education or less; employment status categorised as employed or unemployed; marital status categorised as married or single; monthly household income dichotomized as ≤4000 or >4000 MYR (1000 Malaysian Ringgit about 240 US$); smoking habits, current or former smoker with response options ‘yes’ or ‘no’; and long-term EC usage, with response options ‘yes’ or ‘no’, according to predefined criteria. The second part included 10 items adapted from literature that aimed to evaluate EC user perceptions^25–27^. Items included: getting a definite nicotine hit from e-cigarettes; e-cigarettes are as satisfying as tobacco; e-cigarettes look and feel like a cigarette; e-cigarettes are healthier; e-cigarettes help to cut down smoking; e-cigarettes remove the urge to smoke; the crave for e-cigarettes is as much as for tobacco; use of e-cigarettes in places where smoking is banned; e-cigarettes help to stop smoking; and e-cigarettes allow the use of more nicotine. These items had response options ‘yes’ or ‘no.’

### Data analyses

Data collected were analysed using SPSS version 23.0. Normality check was conducted using statistical and graphical methods for numerical variables, and all numerical data were found to be normally distributed. Descriptive statistics were conducted for all variables. Chi-squared test was used to assess the association between long-term EC usage and categorical variables in this study. Significant variables in the chi-squared test were further examined using binary logistic regression analysis. Odds ratios (ORs) and 95% confidence intervals (CIs) were used to determine the strength of associations between long-term e-cigarette usage and independent variables. We fitted multivariate logistic regression models to examine these associations further. All significant variables at the bivariate level were entered into the multivariate logistic regression analyses to determine the predictors of long-term e-cigarette usage. Adjusted ORs (AORs) and 95% CIs are reported. Statistical significance was set at p<0.05. Multi-collinearity between independent variables was checked.

## RESULTS

### E-cigarette user profiles

Table 1 shows participant EC user profiles. The mean (±SD) age of EC users was 33 (±6.8) years, and the majority were aged >30 years (n=438; 63.1%). Most respondents were Malays (n=608; 87.6%), married (n=473; 68.2%), tertiary educated (n=493; 71%) and employed (n=646; 93.1%) with a monthly household income >4000 MYR (n=301; 43.4%). The mean (±SD) monthly household income was 5850 (±1013) MYR with an income range 100–15000 MYR. Almost all were ever smokers (n=690; 99.4%) while current and former smokers were 10.1% (n=70) and 84.1% (n=584), respectively. The prevalence of long-term EC usage was 82.3%.

**Table 1:** Characteristics of participant e-cigarette users, Malaysia 2018 (N=694)

*Characteristics*	*n (%)*
**Age** (years)	
>30	438 (63.1)
≤30	256 (36.9)
**Ethnicity**	
Malay	608 (87.6)
Non-Malay	86 (12.4)
**Education level**	
Tertiary education	493 (71.0)
Secondary education or less	201 (29.0)
**Employment status**	
Employed	646 (93.1)
Unemployed	48 (6.9)
**Marital status**	
Married	473 (68.2)
Single	221 (31.8)
Monthly household income (MYR)*	
>4000	301 (43.4)
≤4000	393 (56.6)
**Current smoker**	
Yes	70 (10.1)
No	624 (89.9)
**Former smoker**	
Yes	584 (84.1)
No	110 (15.9)
**Long-term e-cigarette usage**	
Yes	571 (82.3)
No	123 (17.7)

* Income level was based on Malaysia’s median household income. MYR: 1000 Malaysian Ringgit about 240 US$.

### E-cigarette user perceptions

Most users reported getting a definite nicotine hit from ECs (n=505; 72.8%). The majority perceived ECs to be as satisfying as tobacco (n=413; 59.5%) and healthier (n=650; 93.7%). Most users believed that ECs have helped them to cut down on tobacco smoking (n=658; 94.8%) and reduce the urge to smoke (n=613; 88.3%). Most respondents believed that ECs have helped them to stop smoking (n=605; 87.2%) (Table 2).

**Table 2:** Perceptions of e-cigarette users, Malaysia 2018 (N=694)

*Perceptions*	*n (%)*
**I get definite nicotine hit from the e-cigarette**	
Yes	505 (72.8)
No	189 (27.2)
**E-cigarette use is as satisfying as tobacco smoking**	
Yes	413 (59.5)
No	281 (40.5)
**I like the e-cigarette because it looks and feels like a cigarette**	
Yes	185 (26.7)
No	509 (73.3)
**E-cigarettes feel healthier than smoking**	
Yes	650 (93.7)
No	44 (6.3)
**The e-cigarette has helped me to cut down tobacco smoking**	
Yes	658 (94.8)
No	36 (5.2)
**I don’t have the urge to smoke as much since using the e-cigarette**	
Yes	613 (88.3)
No	81 (11.7)
**I crave e-cigarettes as much as I do/did for tobacco**	
Yes	246 (35.4)
No	448 (64.6)
**I frequently use the e-cigarette in places where tobacco smoking is banned**	
Yes	96 (13.8)
No	598 (86.2)
**The e-cigarette has helped me to stop smoking**	
Yes	605 (87.2)
No	89 (12.8)
**The e-cigarette allows me to use nicotine more**	
Yes	56 (8.1)
No	638 (91.9)

### Association between long-term e-cigarette usage and user profiles

Table 3 shows the associations between long-term EC usage and user profiles. The odds of being long-term EC users was 2.5 times higher among those aged >30 years (OR=2.5; 95% CI: 1.7–3.3; p<0.001) compared with those aged ≤30 years, and this association was statistically significant. The odds of being long-term EC users was 2.0 times higher among those employed (OR=2.0; 95% CI: 1.1–3.9; p=0.034) compared with those unemployed, and this association was statistically significant. Similarly, the odds of being long-term EC users was 1.7 times higher among those who were married (OR=1.7; 95% CI: 1.1–2.5; p=0.007) compared with singles, and this association was statistically significant. The odds of being long-term EC users was 0.5 times lower among current smokers (OR=0.5; 95% CI: 0.3–0.8; p=0.005) compared with non-current smokers, and this association was statistically significant. In contrast, the odds of being long-term EC users was 2.4 times higher among former smokers (OR=2.4; 95% CI: 1.5–3.7; p<0.001).

**Table 3:** Association between long-term e-cigarette usage and profiles of e-cigarette users, Malaysia 2018 (N=694)

*Characteristics*	*Long-term e-cigarette usage*		*p^a^*	*OR*	*95% CI*	*p*

	*Yes n (%)*	*No n (%)*				
**Age** (years)						
>30	383 (67.1)	55 (44.7)	<0.001*	2.5	1.7–3.3	<0.001*
≤30	188 (32.9)	68 (55.3)				
**Ethnicity**						
Malays	502 (87.9)	106 (86.2)	0.596	1.2	0.7–2.1	0.596
Non-Malays	69 (12.1)	17 (13.8)				
**Education level**						
Tertiary education	412 (72.2)	81 (65.9)	0.162	1.4	0.9–2.0	0.163
Secondary education or less	159 (27.8)	42 (34.1)				
**Employment status**						
Employed	537 (94.0)	109 (88.6)	0.031*	2.0	1.1–3.9	0.034*
Unemployed	34 (6.8)	14 (11.4)				
**Marital status**						
Married	402 (70.4)	71 (57.7)	0.006*	1.7	1.1–2.5	0.007*
Single	169 (29.6)	52 (42.3)				
**Monthly household income** (MYR)						
>4000	257 (45.0)	44 (35.8)	0.061	1.4	0.9–2.0	0.062
≤4000	314 (55.0)	79 (64.2)				
**Current smoker**						
Yes	49 (8.6)	21 (17.1)	0.005*	0.5	0.3–0.8	0.005*
No	522 (91.4)	102 (82.9)				
**Former smoker**						
Yes	494 (86.5)	90 (73.2)	<0.001*	2.4	1.5–3.7	<0.001*
No	77 (13.5)	33 (26.8)				

Predicted outcome category is long-term e-cigarette usage ≥2 years. OR: odds ratio.

* Significant at p<0.05.

a P-value based on chi-squared test. MYR: 1000 Malaysian Ringgit about 240 US$.

### Association between long-term e-cigarette usage and user perceptions

Table 4 shows the associations between long-term EC usage and user perceptions. The odds of being long-term EC users were higher among those who perceived to get a definite nicotine hit from ECs (OR=1.7; 95% CI: 1.1–2.5; p=0.020), felt that ECs were healthier than smoking (OR=2.0; 95% CI: 1.1–3.9; p=0.037), believed that ECs have helped them to cut down tobacco smoking (OR=2.5; 95% CI: 1.2–5.1; p=0.014), perceived that they did not have the urge to smoke as much since using ECs (OR=2.7; 95% CI: 1.6–4.4; p<0.001), and those who perceived that ECs have helped them to stop smoking (OR=3.1; 95% CI: 1.9–5.0; p<0.001) (Table 4). These associations were statistically significant.

**Table 4:** Association between long-term e-cigarette usage and perceptions among e-cigarette users, Malaysia 2018 (N=694)

*Perceptions*	*Long-term e-cigarette usage*		*p^a^*	*OR*	*95% CI*	*p*

	*Yes n (%)*	*No n (%)*				
**I get definite nicotine hit from the e-cigarette**						
Yes	426 (74.6)	79 (64.2)	0.019*	1.7	1.1–2.5	0.020*
No	145 (25.4)	44 (35.8)				
**E-cigarette use is as satisfying as tobacco smoking**						
Yes	345 (60.4)	68 (55.3)	0.293	1.2	0.8–1.8	0.293
No	226 (39.6)	55 (44.7)				
**I like the e-cigarette because it looks and feels like a cigarette**						
Yes	153 (26.8)	32 (26.0)	0.859	1.1	0.7–1.6	0.859
No	418 (73.2)	91 (74.0)				
**E-cigarettes feel healthier than smoking**						
Yes	540 (94.6)	110 (89.4)	0.034*	2.0	1.1–3.9	0.037*
No	31 (5.4)	13 (10.6)				
**The e-cigarette has helped me to cut down tobacco smoking**						
Yes	547 (95.8)	111 (90.2)	0.012*	2.5	1.2–5.1	0.014*
No	24 (4.2)	12 (9.8)				
**I don’t have the urge to smoke as much since using the e-cigarette**						
Yes	517 (90.5)	96 (78.0)	<0.001*	2.7	1.6–4.4	<0.001*
No	54 (9.5)	27 (22.0)				
**I crave e-cigarettes as much as I do/did for tobacco**						
Yes	202 (35.4)	44 (35.8)	0.934	0.9	0.7–1.5	0.934
No	369 (64.6)	79 (64.2)				
**I frequently use the e-cigarette in places where tobacco smoking is banned**						
Yes	75 (13.1)	21 (17.1)	0.251	0.7	0.4–1.2	0.251
No	496 (86.9)	102 (82.9)				
**The e-cigarette has helped me to stop smoking**						
Yes	514 (90.0)	91 (74.0)	<0.001*	3.1	1.9–5.0	<0.001*
No	57 (10.0)	32 (26.0)				
**The e-cigarette allows me to use nicotine more**						
Yes	45 (7.9)	11 (8.9)	0.695	0.8	0.4–1.7	0.695
No	526 (92.1)	112 (91.1)				

Predicted outcome category is long-term e-cigarette usage ≥2 years. OR: odds ratio.

* Significant at p<0.05.

a P-value based on chi-squared test.

### Predictors of long-term e-cigarette usage

Multiple logistic regression analysis yielded two significant predictors of long-term EC usage. The most significant predictor of long-term EC usage in the model was the perception that ECs helped them to stop smoking (AOR=2.5; 95% CI: 1.6–4.4; p<0.001). This was followed by users aged >30 years who had about twice higher odds of being long-term EC users (AOR=2.4; 95% CI: 1.6–3.6; p<0.001). The final model, being the most parsimonious model was statistically significant (χ^2^=41.26; df=3; p<0.001) and predicted 82.3% of long-term EC usage correctly. There was no multi-collinearity between independent variables (Table 5).

**Table 5:** Predictors of long-term e-cigarette usage among e-cigarette users, Malaysia 2018 (N=694)

*Predictors*	*B*	*SE*	*Wald*	Exp (B)*	*95% CI*	*p*
**Age** (years)						
>30	Ref.	Ref.	Ref.	Ref.	Ref.	Ref.
≤30	0.9	0.2	19.3	2.4	1.6–3.6	<0.001
**I get definite nicotine hit from the e-cigarette**						
Yes	0.4	0.2	3.2	1.5	0.9–2.3	0.076
No	Ref.	Ref.	Ref.	Ref.	Ref.	Ref.
**The e-cigarette has helped me to stop smoking**						
Yes	0.9	0.3	14.5	2.5	1.6–4.4	<0.001
No	Ref.	Ref.	Ref.	Ref.	Ref.	Ref.

Variables entered: All significant variables in bivariate analysis (Regression analysis conducted based on Backward Wald technique).

* Exp (B) gives the adjusted odds ratio (AOR). ant at p<0.05.

## DISCUSSION

Long-term EC users constituted 82.3%. Most users believed that ECs have helped them to cut down on tobacco smoking, reduced the urge to smoke, and ultimately helped them to stop smoking. Respondents aged >30 years and those who perceived that ECs have helped them to stop smoking were significantly more likely to be long-term EC users.

### Study population

The profile of EC users in this study was consistent with international and local trends, albeit with some notable differences. Vaping has become progressively popular among adolescents and young adults, worldwide, and the duration of EC use is believed to vary depending on whether usage is for experimental (e.g. curiosity) or goal-oriented (quit-smoking) reasons^18,28,29^. The average age of EC users in the current study (33 years) was considerably higher than that reported in previous studies in Malaysia^19,23^. This is probably due to differences in the design of the current and previous studies. While the current study targeted EC users across Malaysia, the earlier studies were state-focused and estimated prevalence of EC use among the general public. It is also possible that the self-selecting nature of participants in our sample reflects a higher participation by those who strongly identify as current regular users of e-cigarettes. To this extent, it seems reasonable to assume that this type of goal-oriented usage is concentrated in the older population, and this might have influenced the direction of our study findings.

Consistent with a previous report by Wong et al.^19^, EC users in the current study were predominantly Malays, married, and attained higher than secondary education. Compared to the earlier studies, a higher proportion of EC users in the current study were employed and earned a higher monthly household income.

The representation of participants by state in this study approximates the national population distribution, albeit with slight differences, with about one-third of respondents in the current study being from Selangor (including Putrajaya), which is the most populous and economically vibrant state in Malaysia as at 2019^30^. Consistent with the national population distribution, respondents from Perlis were the least represented in our study.

### E-cigarette user perceptions

Findings from the current study were in accordance with earlier reports regarding perceived benefits of EC use^31^. There is rich literature evidence supporting the use of ECs as a smoking cessation aid^32^, although the extent to which it helps smokers to completely quit smoking is still a subject of debate^33^. Consistent with earlier studies, participants in the current study believed that ECs have helped them to cut down on tobacco smoking^34,35^, reduced the urge to smoke^36–38^ and ultimately helped them to stop smoking. While its benefit as a smoking cessation aid remains the most common reason for long-term EC usage among our study participants and vapers around the world, there are arguments regarding its role or efficacy in nicotine cessation^39,40^, which, according to a recent systematic review, cast doubt on its overall efficacy in smoking cessation^41^. Nevertheless, the potential benefits that ECs offer over traditional cigarettes in preserving lung and other metabolic functions and reducing indoor and outdoor air pollution remain of interest to many stakeholders including public health practitioners and policy makers.

### Predictors of long-term e-cigarette usage

We found in this study that those aged >30 years and the perception among vapers that ECs have helped them to quit smoking were significant predictors of long-term EC usage. This is perhaps not surprising, particularly amongst the predominantly goal-oriented EC users in our study cohort. Previous studies have shown that although nicotine addiction remains a key driver of EC use, other factors such as being more economical and environment-friendly, public policy acceptance, and being less harmful to health, were considered strong reasons for continuous and long-term EC usage among vapers in Malaysia and other parts of the world^6,19,42,43^. Although we did not expressly collect data on the age at onset of EC use among participants in this study, the fact that most respondents engage in its use as a smoking cessation aid extends the argument that they possibly have been using it over years and may continue to do so for a long time. Our data on proportion of current smokers (10%) relative to former smokers (84%) among EC users in the current study further suggests that not only they may have been engaging in EC use for smoking cessation in the short-term, but also there is the possibility that they would continuously engage in its use over the long-term to avoid relapse and to attenuate cravings. This finding is consistent with that of a previous study that suggested that long-term EC usage was associated with a higher rate of smoking cessation^15^. Also, Zhuang et al.^15^ reported a higher likelihood of current EC users becoming long-term EC users, and that higher smoking cessation rates are associated with intensity of EC use (daily use)^15^. Combined, the findings of the current study that older, goal-oriented EC users were more likely to use ECs in the long-term have a sound basis in literature since the pharmacotherapeutic effect of smoking cessation aids in the real-world setting can only be detected if they are used for a sufficient long period of time^15,44^.

### Strengths and limitations

One of the major strengths of this study is the recruitment and participation of a nationwide sample of EC users across all states in Malaysia, improving the internal and external validity of the findings as well as its comparability with other similar studies conducted internationally. To the best of our knowledge, this is the first nationwide survey that explored the factors associated with long-term EC usage among vapers in Malaysia, and this provides a platform upon which future strategic interventions including public policies could built on.

The participants in this survey required access to online computer technology and thus our recruitment path may have been weighted not only towards those in contact with active long-term EC use peers, but also those of higher socioeconomic status. Participants were recruited from users registered in an association of EC users, and thus they may be expected to give ‘positive’ feedbacks about EC use as a smoking cessation aid, resulting in social desirability bias. According to the Malaysian Poisons Act 1952, the sale of ECs with nicotine content is illegal, unless prescribed by licensed health professionals^45^. The lack of comparison perceptions among similar smokers who are not currently using ECs is another weakness and limits the full picture of the role that ECs may have in the lives of Malaysian smokers. From surveys elsewhere, one would assume that the perceptions of those smokers not currently using ECs are likely to be substantially different from those who are current users. They are more likely to believe that they are harmful and that they are not a useful substitute for tobacco smoking. This has further implications for harm reduction policy^46^. Although user perceptions were well demonstrated in the current study, this online survey could not establish veracity of smoking abstinence through biochemical assessment. Thus, future studies should be directed towards verifying smoking abstinence through biochemical assessments in long-term EC users.

## CONCLUSIONS

Malaysians generally engage in EC use as a smoking cessation aid. This reflects their perception that ECs have helped them to cut down on tobacco smoking, reduced the urge to smoke and ultimately helped them to stop smoking. They were more likely to be long-term e-cigarette users if they were older (>30 years) and perceived that e-cigarette use helped them to quit smoking. This is consistent with the growing body of evidence on the effectiveness of ECs as a smoking cessation aid at population level, as well as global consensus about ECs being less harmful than conventional tobacco smoking, a view shared by most of our respondents. These findings provide important information on EC users profile nationwide, a strong basis for targeted prevention, health promotion and smoking cessation interventions, and policy thrust for EC regulation in Malaysia.
